# Menopause-related brain fog as a midlife window in women's brain aging: toward ecologically valid measurement and digital phenotyping

**DOI:** 10.3389/fnhum.2026.1814092

**Published:** 2026-04-21

**Authors:** Parisa Gazerani

**Affiliations:** 1 Department of Life Sciences and Health, Faculty of Health Sciences, Oslo Metropolitan University, Oslo, Norway; 2 Advanced Health Intelligence and Brain-Inspired Technologies (ADEPT), Faculty of Technology, Art and Design, Oslo Metropolitan University, Oslo, Norway

**Keywords:** aging neuroscience, brain fog, cognitive complaints, digital phenotyping, ecological momentary assessment, menopause, wearables, women's brain health

## Abstract

Cognitive complaints commonly described as “brain fog” are frequent during the menopause transition and often involve attention and memory difficulties that can affect daily functioning and quality of life. Midlife women may worry these symptoms signal early neurodegenerative disease, yet available evidence indicates that menopause-related cognitive changes are typically mild, variable, and distinct from dementia. Midlife, typically spanning approximately ages 40–60 years and encompassing the menopause transition (most commonly occurring between 45–55 years), represents a critical period for women's brain health. A major barrier to progress is that “brain fog” remains inconsistently defined and measured, limiting comparability across studies and constraining prevention-oriented strategies. In this Perspective, we propose that menopause-related brain fog represents a time-limited, clinically meaningful “measurement window” in women's brain aging trajectories, in which symptoms may be most detectable and potentially modifiable if assessed with tools that capture real-world fluctuation. We propose a pragmatic, multi-layer measurement framework that integrates (1) patient-centered symptom and functional impact profiling, (2) brief targeted cognitive assessment of vulnerable domains, and (3) ecological momentary assessment combined with passive and active digital measures using wearable and smartphone-based metrics (sleep, activity/circadian regularity, autonomic proxies, and brief repeated digital cognitive tasks). We outline validation principles, equity considerations, and reporting recommendations aligned with sex- and gender-aware research practices. By operationalizing menopause-related brain fog and linking it to feasible digital measurement strategies, aging neuroscience can better distinguish normative midlife cognitive variability from concerning trajectories, accelerate mechanistic research, and support preventive brain health interventions for women.

## 1 Introduction

Aging neuroscience has progressively shifted from a disease-centered framework toward a lifespan perspective emphasizing resilience, maintenance of function, and prevention of decline (Fjell, 2024). Rather than viewing aging solely through the lens of neurodegenerative disorders, contemporary models increasingly conceptualize aging as a dynamic process shaped by biological transitions, environmental exposures, behavioral adaptation, and cognitive reserve accumulated across life (Asejeje and Ogunro, 2024). Within this framework, identifying transitional periods during which brain function becomes temporarily more sensitive to internal and external influences represents a major opportunity for preventive neuroscience and early intervention research (Tenchov et al., 2024). In this context, it is important to distinguish between key terms. Midlife refers to a broader life stage encompassing the years before and after menopause. Perimenopause denotes the transitional phase leading up to the final menstrual period, characterized by hormonal fluctuation, while menopause is defined retrospectively after 12 consecutive months without menstruation. The term menopause transition is used here to capture this broader dynamic period.

Women's aging trajectories include physiological transitions that are both biologically distinctive and historically underrepresented in neuroscience research (Warren et al., 2025). The menopause transition represents one such period. Longitudinal cohort studies, including findings from the Study of Women's Health Across the Nation (SWAN), demonstrate that subjective cognitive complaints often described as brain fog are common during midlife and may coincide with modest, domain-specific changes in cognitive performance, particularly processing speed and verbal memory, without indicating neurodegenerative disease (Greendale et al., 2010; El Khoudary et al., 2019).

Many midlife women report symptoms including forgetfulness, reduced concentration, slowed thinking, distractibility, and word-finding difficulty (Metcalf et al., 2023). Although these symptoms are typically mild, they can meaningfully affect occupational functioning, daily activities, and self-confidence. Importantly, cognitive complaints during midlife frequently generate anxiety about impending dementia, despite clinical evidence indicating that menopause-related cognitive changes are generally transient and distinct from progressive cognitive decline associated with neurodegenerative disease (Maki and Jaff, 2022; Conde et al., 2021).

The scientific challenge lies not only in determining whether cognitive changes occur, but in how they are conceptualized and measured (Conde et al., 2021; Maki and Thurston, 2020). The term “brain fog” is widely used across clinical and public discourse, yet lacks standardized operational definitions or measurement frameworks (Lucius, 2021). As a result, findings remain difficult to compare across studies, and clinicians face uncertainty when counseling patients. Traditional cognitive assessment paradigms, typically based on single clinic visits and global screening instruments, were developed to detect stable deficits rather than fluctuating changes and are therefore poorly suited to capture context-dependent cognitive experiences unfolding in everyday life (Maki and Jaff, 2022; Conde et al., 2021; Durakli Ulukök et al., 2024).

From an aging neuroscience perspective, menopause may represent a unique midlife window in which cognitive variability becomes particularly observable (Henderson, 2011; Ramli et al., 2023). The menopause transition interacts with sleep disruption, vasomotor symptoms, stress physiology, mood changes, and competing life demands characteristic of midlife, potentially amplifying day-to-day variability in attention and memory processes (Lang et al., 2025; Gava et al., 2019). Increasing evidence from cognitive aging research suggests that intra-individual variability itself may provide important insight into brain function and adaptive capacity, sometimes revealing meaningful change even when average performance remains stable (Mohr and Nagel, 2010; Salthouse, 2007; Brydges et al., 2020).

Rather than interpreting such variability solely as impairment, it may instead reflect adaptive neurobiological recalibration occurring during a time-limited transition. This perspective reframes menopause-related cognitive complaints as an opportunity to study dynamic brain–behavior relationships under naturalistic conditions (Devi, 2018; Bangle et al., 2025).

In this Perspective, menopause-related brain fog is reframed as a measurement opportunity in women's brain aging. Advancing the field requires moving beyond cross-sectional cognitive testing toward ecologically valid approaches that capture within-person fluctuations in brain fog symptoms and subjective cognitive experience in real-world contexts. Here, therefore, a pragmatic, multi-layer measurement framework is outlined, integrating patient-centered symptom profiling, targeted cognitive assessment, and ecological momentary assessment combined with wearable and smartphone-based digital phenotyping. By operationalizing menopause-related brain fog in this way, aging neuroscience may better distinguish normative variability from concerning trajectories, generate testable mechanistic hypotheses, and support preventive brain health strategies tailored to midlife women.

Unlike prior work focusing on whether menopause causes cognitive decline, this Perspective reframes menopause as a biologically time-limited measurement window enabling observation of within-person cognitive dynamics using ecologically valid methods. Importantly, this Perspective focuses on menopause-related subjective cognitive complaints (brain fog) rather than on long-term dementia risk, aiming to better characterize fluctuating cognitive experiences in midlife and distinguish them from progressive neurodegenerative trajectories.

## 2 Is brain fog “a woman's condition”?

Brain fog is not exclusive to women, nor is it unique to menopause (Johnson and Ogden, 2025). Similar subjective cognitive complaints have been described across a wide range of clinical and non-clinical contexts, including chronic pain disorders, autoimmune disease, post-viral syndromes, sleep disturbances, mood disorders, and conditions characterized by sustained fatigue or physiological stress (McWhirter et al., 2023; Denno et al., 2025; Dass et al., 2023; Azcue et al., 2022). Across these settings, individuals frequently report reduced mental clarity, attentional lapses, slowed thinking, and memory difficulties that fluctuate over time and are not always reflected in objective cognitive testing, including brief cognitive screening tests. The recurrence of this experiential pattern across conditions suggests that brain fog reflects a broader neurofunctional phenomenon rather than a disorder tied to a single disease entity (Hill et al., 2022).

From a neuroscience perspective, symptom clusters such as fatigue, pain, and perceived cognitive dysfunction are increasingly recognized as meaningful expressions of brain–body interaction rather than nonspecific complaints (Lin, 2023). Evidence from chronic pain and post-viral research indicates that subjective cognitive dysfunction may arise from interactions among sleep disruption, stress physiology, attentional demand, autonomic regulation, and affective state, all of which influence cognitive efficiency in real-world environments (Azcue et al., 2022; Zerbini et al., 2025; Patel et al., 2025; Ocon, 2013). Notably, these symptoms frequently demonstrate substantial intra-individual variability, underscoring the limitations of single time-point cognitive assessments and supporting the need for ecologically valid measurement approaches (Furey et al., 2025; Zhu et al., 2025; Vigliocco et al., 2024).

Within this broader transdiagnostic landscape, menopause provides a distinct and historically understudied biological–behavioral context with direct relevance to women's aging neuroscience (El Khoudary et al., 2019; Crestol et al., 2023). The menopause transition is characterized by dynamic endocrine changes accompanied by vasomotor symptoms, sleep fragmentation, mood fluctuations, and alterations in thermoregulation and stress responsivity (Greendale et al., 2010; Santoro et al., 2021). Cognitive complaints are frequently reported during this period, with longitudinal studies demonstrating modest but detectable changes particularly in verbal learning and memory and, to a lesser extent, attention and working memory (Conde et al., 2021; Epperson et al., 2013). Importantly, these changes typically occur alongside symptom burden (i.e., the cumulative impact of co-occurring symptoms such as sleep disturbance, vasomotor symptoms, mood changes, and fatigue) (Maki and Jaff, 2022; Maki and Thurston, 2020) rather than reflecting progressive neurodegenerative pathology (Zuhlsdorff et al., 2026).

A critical clinical consideration is that dementia during midlife remains uncommon (Fletcher, 2021; Gandy et al., 2017; Li et al., 2026). Nevertheless, subjective cognitive symptoms during menopause often carry disproportionate emotional significance, as women may interpret lapses in memory or concentration as early indicators of irreversible decline (Conde et al., 2021; Zhu et al., 2025; Choi et al., 2025). This mismatch between perceived cognitive symptoms and underlying biological risk does not indicate current or imminent neurodegenerative pathology, but rather reflects differences between subjective experience, contextual influences, and objective measurement. It may contribute to increased help-seeking behavior and uncertainty in clinical interpretation (Zhu et al., 2025; Liew, 2020). Addressing menopause-related brain fog therefore has relevance not only for neuroscience research but also for clinical communication, reassurance, and preventive brain health strategies.

Menopause-related brain fog can therefore be conceptualized along three complementary dimensions:

- Transdiagnostic in concept: subjective cognitive dysfunction occurs across multiple conditions and reflects shared neurofunctional mechanisms involving sleep, stress regulation, and cognitive load (including both working memory demands and broader psychosocial and emotional demands).
- Sex-specific in timing and drivers: the menopause transition represents a unique endocrine and physiological context in which interacting symptoms may transiently alter cognitive experience.
- Women-specific in clinical meaning: cognitive complaints during midlife frequently trigger dementia-related fears and influence help-seeking behavior, shaping interpretation and management of symptoms.

This framing avoids positioning brain fog as a novel disease entity while recognizing it as a clinically meaningful and scientifically tractable phenomenon. It also aligns with sex- and gender-aware research principles by acknowledging biological specificity without excluding broader transdiagnostic insights (Johnson and Ogden, 2025). Situating menopause-related brain fog within this framework enables aging neuroscience to move beyond binary distinctions between “normal aging” and disease and toward understanding how transitional life stages modulate cognition in real-world contexts.

## 3 Why some women and not others? A layered vulnerability model

A defining feature of menopause-related brain fog is its heterogeneity (Maki and Jaff, 2024). While many women report transient cognitive changes during the menopause transition, others experience minimal symptoms, and a smaller subgroup reports persistent or functionally significant difficulties. Longitudinal cohort studies from the Study of Women's Health Across the Nation (SWAN) demonstrate that cognitive changes during perimenopause (the transitional phase preceding menopause) are modest, variable, and often reversible after the transition, supporting the view that menopause alone is unlikely to be a sufficient explanatory factor (Greendale et al., 2010, 2009).

Instead, menopause-related brain fog may emerge from interactions between endocrine transition and pre-existing biological, psychological, and lifestyle-related vulnerabilities that shape cognitive resilience across midlife (Conde et al., 2021; Maki and Jaff, 2024). A layered vulnerability framework, therefore, provides a useful conceptual model, recognizing that menopause-related cognitive complaints arise from converging influences operating across neurobiological, behavioral, and contextual levels rather than a single causal pathway. Together, these interacting systems suggest that menopause may transiently alter neural efficiency and regulatory stability rather than structural integrity, providing a plausible basis for increased cognitive variability.

### 3.1 Neuroendocrine transition and brain sensitivity

The menopause transition involves fluctuations and eventual decline in ovarian hormones, particularly estradiol, which plays important modulatory roles in synaptic plasticity, neurotransmission, and cerebral energy metabolism. Estrogen receptors are widely distributed in brain regions critical for cognition, including the hippocampus and prefrontal cortex, supporting roles in memory and executive regulation (Shanmugan and Epperson, 2014; Russell et al., 2019).

Evidence indicates that cognitive performance does not change linearly across menopause; instead, transient reductions in verbal learning, attention, and processing efficiency may occur during perimenopause before stabilizing post-menopause (Weber et al., 2013).

Importantly, interindividual differences in sensitivity to hormonal fluctuation appear substantial. Contemporary models, therefore, increasingly view endocrine transition as a contextual amplifier that increases vulnerability to other physiological and psychological stressors, rather than as a direct cause of AD/ADRD-related pathology (Maki and Thurston, 2020). These pathways represent an active area of research, and current evidence suggests complex, multifactorial relationships rather than direct causal links.

### 3.2 Sleep disruption and thermoregulatory instability

Sleep disturbance represents one of the most consistent correlates of menopause-related cognitive complaints. Vasomotor symptoms and nocturnal awakenings commonly fragment sleep continuity, and SWAN data demonstrate associations between sleep disruption and reduced cognitive processing efficiency during midlife (El Khoudary et al., 2019).

Sleep research consistently shows that even modest sleep restriction impairs attention, working memory, and executive functioning the following day, indicating that stable sleep is necessary for normal cognitive functioning (Wüst et al., 2024). Because sleep quality varies night to night (Suh et al., 2012), it likely contributes directly to the fluctuating nature of brain fog symptoms rather than reflecting stable cognitive impairment.

Within this framework, menopause-related cognitive complaints may often reflect downstream consequences of sleep instability, highlighting sleep regulation as a potentially modifiable driver of perceived cognitive decline.

### 3.3 Stress physiology, mood, and cognitive load

Midlife frequently coincides with increased psychosocial demands, including occupational responsibilities, caregiving roles, and health-related stressors (Infurna et al., 2020). Changes in stress responsivity and mood symptoms during menopause may influence cognition through altered attentional allocation and increased perceived mental effort (Weber et al., 2014). Subjective cognitive complaints commonly correlate with symptom burden even when objective performance changes are small, illustrating how stress and affective state shape cognitive appraisal (Furey et al., 2025). Importantly, subjective cognitive difficulty should not be interpreted as absence of neurobiological change; rather, it may indicate increased neural effort required to maintain performance under fluctuating physiological conditions (Wei et al., 2022).

### 3.4 Cognitive reserve and life-course factors

Life-course influences including education, occupational complexity, physical activity, and social engagement contribute to cognitive reserve, shaping resilience to physiological stressors during aging (Mendoza-Ruvalcaba et al., 2026; Frank et al., 2023). Cognitive reserve refers to the capacity of the brain to maintain function despite physiological or pathological challenges and is shaped by life-course factors such as education and engagement (Stern and Barulli, 2019). Aging neuroscience increasingly recognizes reserve as a moderator of how individuals experience transitional brain states rather than solely determining late-life decline (Pappalettera et al., 2024; Cheng, 2016). Midlife cohort studies demonstrate substantial interindividual variability in cognitive trajectories, suggesting that reserve-related compensatory mechanisms may allow some women to maintain stable functioning despite hormonal and symptomatic change (Karlamangla et al., 2017). This perspective aligns menopause-related brain fog with adaptive models of aging emphasizing flexibility and compensation rather than deficit alone.

### 3.5 Comorbid symptom domains

Conditions frequently co-occurring in midlife, including chronic pain, migraine, metabolic dysregulation, and mood disorders, may further modulate cognitive experience (Yu et al., 2026; Choudhary, 2024). Research across fatigue and chronic pain populations shows that overlapping symptom networks influence attentional control, perceived mental clarity, and participation in everyday activities (Peterson et al., 2023). Menopause-related brain fog may therefore represent a convergence point in which multiple symptom systems interact, rather than an isolated cognitive phenomenon confined to endocrine change (Conde et al., 2021).

Taken together, this layered model reframes menopause-related brain fog as the outcome of interacting vulnerabilities rather than a uniform consequence of hormonal decline. Such a perspective helps explain individual variability and provides a conceptual foundation for personalized measurement strategies. Importantly, it also supports a prevention-oriented framework: if symptoms arise from modifiable interacting drivers, particularly sleep disruption, stress burden, and lifestyle factors, then identifying these relationships through longitudinal and ecologically valid measurement may enable targeted intervention during a critical midlife window.

## 4 The core challenge: definition and measurement

A central barrier to progress in understanding menopause-related brain fog is not the absence of reported symptoms, but the absence of shared conceptual and measurement frameworks. Across clinical and research contexts, the term brain fog is widely used to describe subjective cognitive inefficiency, yet its meaning varies substantially between disciplines, study populations, and methodological traditions. Without operational definitions that link lived experience to measurable domains, cumulative knowledge cannot develop and clinical interpretation remains inconsistent (Maki and Jaff, 2022; Dass et al., 2023).

This problem reflects a broader limitation within aging neuroscience. Conventional cognitive assessment paradigms were largely designed to detect stable deficits associated with neurological disease and therefore emphasize single time-point testing under controlled laboratory conditions and between-person comparisons (Caine and Grossman, 1992). However, menopause-related cognitive complaints are characterized primarily by variability rather than decline. Symptoms fluctuate across days and contexts, influenced by sleep quality, vasomotor symptoms, emotional state, stress exposure, and competing cognitive demands. Consequently, traditional assessment approaches may fail not because symptoms are absent, but because measurement tools are misaligned with the phenomenon under investigation (Hultsch et al., 2008; Spooner and Pachana, 2006).

Two interrelated measurement paradoxes therefore define menopause-related brain fog:

*Subjective–objective mismatch:*

First, a subjective–objective mismatch is frequently observed. Women may report substantial cognitive difficulty despite performance within normative ranges on screening tests, while subtle domain-specific inefficiencies may remain undetected when assessment relies solely on global cognitive measures. Similar dissociations between perceived and measured cognition have been documented in subjective cognitive decline, chronic pain, and fatigue research, suggesting that subjective experience reflects cognitive effort, confidence, and functional participation rather than accuracy alone (Dass et al., 2023; Rabin et al., 2015). This mismatch should therefore not be interpreted as evidence that symptoms are purely psychological. Instead, it highlights a distinction between optimized laboratory performance and cognitive functioning as experienced in everyday environments.

*Temporal dynamics and fluctuation:*

Second, menopause-related brain fog is inherently dynamic. Cognitive efficiency may vary substantially from 1 day to the next depending on sleep fragmentation, thermoregulatory disturbance, mood fluctuations, workload, or stress exposure. Growing evidence in cognitive aging indicates that intra-individual variability itself may represent an informative marker of neural regulation and adaptive capacity rather than measurement noise (Hultsch et al., 2008; MacDonald et al., 2009). Cross-sectional clinic snapshots are therefore insufficient to capture the temporal structure of symptoms.

In this Perspective, menopause-related brain fog is defined as a fluctuating cluster of subjective cognitive complaints occurring during the menopause transition that produces measurable functional impact. Symptoms most commonly involve attention, working memory, or verbal memory complaints and frequently co-occur with sleep disturbance, vasomotor symptoms, and mood or stress changes. Importantly, this construct represents a functional syndrome rather than a diagnosis, and assessment should support reassurance and appropriate follow-up rather than pathologization (Maki and Jaff, 2022). For research and early clinical piloting, three provisional phenotypes may help structure heterogeneity:

- State-like brain fog: episodic, context-dependent symptoms strongly associated with sleep loss, stress, or acute symptom burden.
- Symptom-burden brain fog: cognitive complaints emerging alongside sustained vasomotor symptoms and chronic sleep disruption.
- Persistent brain fog: relatively stable symptoms or increasing functional impact warranting broader clinical evaluation.

These phenotypes are not mutually exclusive but provide a pragmatic starting framework for stratification and hypothesis generation.

To translate these conceptual distinctions into research practice, menopause-related brain fog requires an operational framework that links subjective experience, measurable cognitive domains, functional consequences, and contextual drivers. Table 1 summarizes how menopause-related brain fog can be operationalized across complementary measurement domains, linking subjective experience, objective performance, and contextual drivers within an ecologically valid framework. Ecological momentary assessment (EMA), defined as repeated real-time self-reports in daily life contexts, enables capture of within-person variability central to this framework (Stinson et al., 2022).

**Table 1:** Conceptual and measurement framework for operationalizing menopause-related brain fog.

Construct	Working definition	Observable indicators	Measurement approach
Menopause-related brain fog	Fluctuating subjective cognitive inefficiency during menopause transition with functional impact	Mental cloudiness, distractibility, memory slips, slowed thinking	Symptom profiling + repeated assessment
Subjective cognitive experience	Perceived difficulty independent of single-test outcomes	Self-rated clarity, effort, forgetfulness incidents	EMA, brief questionnaires
Objective cognitive efficiency	Performance in vulnerable domains	Attention, working memory, verbal learning, processing speed	Brief repeatable cognitive tasks
Functional impact	Effects on participation and daily activities	Work performance, medication management, task completion	Functional scales, diary
Contextual drivers	Modulators of cognitive variability	Sleep disruption, vasomotor symptoms, stress, pain	Wearable sleep, EMA context tracking

EMA, Ecological Momentary Assessment.

## 5 Menopause-related brain fog as a midlife measurement window

The menopause transition represents a biologically time-limited yet symptom-rich phase that offers a unique opportunity for aging neuroscience (Mosconi et al., 2021). Rather than viewing cognitive complaints solely as a clinical concern, menopause may be understood as a natural observational period and a measurement opportunity for understanding adaptive brain regulation rather than pathologizing normal experience.

Longitudinal cohort evidence indicates that menopause amplifies fluctuations in brain fog symptoms and subjective cognitive experience rather than reflecting progressive cognitive decline associated with neurodegenerative disease (Maki and Jaff, 2022; Greendale et al., 2009). At the same time, research in cognitive aging increasingly recognizes intra-individual variability as an informative marker of neural regulation and adaptive capacity rather than measurement noise (Hultsch et al., 2008; MacDonald et al., 2009).

From this perspective, menopause functions as a measurement window: a period in which relationships between everyday physiology and cognition can be examined with unusual sensitivity. Hormonal fluctuation coincides with sleep disruption, vasomotor instability, and changing stress responsivity, temporarily increasing the visibility of within-person cognitive variability. Studying cognition during this window therefore allows aging neuroscience to move beyond static deficit detection toward understanding dynamic regulation of brain function in real-world environments.

Capturing this opportunity requires a shift from cross-sectional assessment toward longitudinal and ecologically grounded observation capable of integrating subjective experience, repeated cognitive performance, and contextual physiological signals.

In short, the menopause transition is conceptualized as a time-limited biological and psychosocial context in which hormonal fluctuation, sleep disruption, vasomotor symptoms, stress, and midlife demands amplify physiological–cognitive interactions. This process creates a midlife measurement window characterized by increased within-person cognitive variability across subjective experience, cognitive performance, and daily contextual factors. Ecologically grounded assessment, including EMA, wearable monitoring, and brief digital cognitive tasks, enables longitudinal characterization of this variability and identification of modifiable drivers. These insights support prevention-oriented and personalized brain health strategies while clinical interpretation is guided by validation, methodological rigor, equity, and responsible implementation.

## 6 Operationalizing ecologically valid measurement

Traditional neuropsychological assessment was designed to detect stable impairment under controlled conditions. While essential for diagnosing neurological disease, such approaches are poorly suited to phenomena characterized by fluctuation and context dependence, where perceived cognitive dysfunction often emerges despite preserved performance under optimized testing environments (Dass et al., 2023; Rabin et al., 2015; Hampshire et al., 2021).

Ecologically valid measurement, therefore, requires integrating multiple complementary layers of observation. First, structured profiling of subjective experience captures perceived clarity, effort, and functional consequences that are invisible to performance scores alone. Second, brief repeatable cognitive tasks targeting attention, working memory, and processing speed allow assessment of intra-individual variability across time rather than reliance on single measurements. Third, ecological momentary assessment and wearable sensing enable contextualization of cognition within daily physiology, including sleep continuity, circadian regularity, and stress-related physiological signals.

Importantly, the goal of digital measurement is not diagnostic classification. Instead, these approaches allow modeling of within-person relationships between symptoms and modifiable drivers, an approach aligned with emerging precision health and N-of-1 methodologies (Insel, 2018; Huckvale et al., 2019). Short-term “measurement bursts,” combining repeated cognitive micro-tasks with ecological monitoring over two- to four-week intervals, provide a feasible strategy for capturing temporal dynamics while minimizing participant burden.

When integrated, these methods enable researchers to distinguish normative variability from emerging vulnerability and to generate individualized hypotheses regarding mechanisms linking sleep, stress regulation, and cognitive efficiency. Table 2 provides examples of ecologically valid measurement paradigms and illustrates how these approaches translate into testable hypotheses and preventive strategies.

**Table 2:** Ecologically valid measurement paradigms illustrating testable hypotheses and preventive applications.

Paradigm	Data captured	Example hypothesis	Preventive or clinical implication
EMA combined with wearable sleep monitoring	Repeated self-reported cognitive clarity, fatigue, and sleep continuity metrics	Reduced sleep continuity predicts next-day increases in perceived brain fog severity	Sleep-focused behavioral or clinical intervention
Brief digital cognitive micro-tasks	Within-person variability in attention, reaction time, and working memory performance	Performance variability reflects contextual and physiological influences rather than fixed deficit	Optimization of assessment timing and workload management
Longitudinal “measurement bursts”	Repeated multimodal monitoring across defined time windows	Symptom variability decreases following targeted intervention	Individualized preventive strategy development
N-of-1 monitoring designs	Alternating behavioral or lifestyle interventions with continuous tracking	Within-person changes identify modifiable drivers of cognitive symptoms	Personalized care pathways and adaptive intervention planning

EMA, Ecological Momentary Assessment.

## 7 Validation principles for ecologically valid cognitive measurement

Integrating EMA, wearable sensing, and brief digital cognitive testing into aging neuroscience introduces both opportunity and methodological responsibility. Digital approaches provide access to real-world cognitive variability, but their scientific value depends on rigorous validation and cautious interpretation. Without clear methodological standards, digital phenotyping risks being perceived as technologically appealing yet scientifically uncertain (Insel, 2018; Huckvale et al., 2019).

Digital measures should therefore be understood as measurement augmentation rather than diagnostic replacement. Their purpose is not to substitute clinical evaluation or standardized neuropsychological testing, but to extend observation into everyday environments where cognitive challenges occur.

Validation requires convergent and ecological evidence. Associations between EMA-reported cognitive clarity, functional outcomes, and targeted cognitive performance should demonstrate patterned relationships rather than perfect correspondence, reflecting the distinction between perceived effort and task accuracy documented in subjective cognitive decline research (Rabin et al., 2015). Equally important is ecological validity: variability arising from time-of-day, interruptions, or workload represents meaningful signal rather than measurement error. Because menopause-related brain fog is fundamentally a within-person phenomenon, analytic approaches should prioritize intra-individual dynamics over between-person comparisons. Longitudinal modeling linking sleep disruption, stress exposure, or symptom burden to next-day cognitive experience aligns with emerging precision-health frameworks emphasizing temporal processes (Wang et al., 2018).

Careful management of confounding variables, including education, mood symptoms, sleep disorders, medication use, chronic pain, and hormone therapy, is essential to avoid overattributing findings to menopause alone. Physiological proxies derived from wearable devices should likewise be interpreted cautiously, as heart-rate variability and sleep staging algorithms vary across devices and contexts. Where feasible, hybrid validation designs comparing digital metrics with established reference standards (e.g., actigraphy, polysomnography, standardized neuropsychological testing) can strengthen interpretability while preserving scalability. Transparent reporting of devices, sampling frequency, adherence, and analytic decisions remains essential for reproducibility and cumulative progress.

Together, these principles position ecologically grounded measurement as part of an evolving methodological ecosystem aimed at characterizing cognition as a dynamic process embedded in daily life rather than a static laboratory outcome.

## 8 Responsible implementation, clinical translation, and future directions

The growing use of digital measurement approaches in aging neuroscience introduces both scientific opportunity and ethical responsibility (Insel, 2018; Huckvale et al., 2019). Wearable technologies, ecological momentary assessment, and repeated digital cognitive testing offer scalable methods for studying cognition in real-world contexts; however, their implementation must be guided by considerations of equity, interpretability, and long-term sustainability.

Menopause-related brain fog provides a particularly relevant context in which to address these challenges. Midlife women represent a highly heterogeneous population with respect to socioeconomic background, occupational demands, caregiving responsibilities, and access to healthcare. Research designs relying exclusively on costly devices or technologically complex interfaces risk privileging highly educated or digitally confident participants, thereby limiting generalizability and reinforcing sampling biases already recognized in cognitive aging research (Veinot et al., 2018). Inclusive design should therefore be viewed as a methodological requirement rather than an ethical afterthought. Low-burden participation options, including simplified wearable devices, SMS-based ecological assessments, and flexible reporting schedules, can broaden accessibility while maintaining scientific rigor.

Responsible digital research must also address privacy and data governance. Continuous monitoring of sleep, activity, or behavioral routines generates sensitive personal information, making transparent communication regarding data use, storage, and participant autonomy essential for maintaining trust (Nebeker et al., 2019). Incorporating participant perspectives through co-design approaches may further improve acceptability while ensuring that measurement prioritizes outcomes meaningful to women themselves, such as work functioning, confidence, and participation in daily life.

Clinically, reframing menopause-related brain fog as a measurable and context-sensitive phenomenon may help shift conversations away from binary interpretations of “normal aging” vs. disease. Many women seek reassurance during midlife cognitive change, yet clinicians often lack tools to distinguish transient variability from patterns warranting further evaluation (Maki and Jaff, 2022). Structured symptom profiling combined with short-term longitudinal monitoring may support more nuanced counseling, allowing normalization of common experiences while preserving attention to potential red flags.

From a research perspective, menopause-related brain fog highlights the importance of within-person variability as a meaningful signal of brain regulation (MacDonald et al., 2009). Studying cognitive fluctuation alongside sleep quality, vasomotor symptoms, emotional state, and daily context may reveal mechanisms linking physiological regulation to cognitive efficiency across the lifespan. Operationalizing menopause-related brain fog as a measurable phenotype may also facilitate harmonization across studies by establishing shared outcome domains, enabling cumulative datasets and meta-analytic synthesis across menopause science, fatigue research, chronic pain, and stress-related cognitive change.

A central promise of ecologically valid measurement lies in its potential to translate observation into prevention. Longitudinal monitoring allows movement beyond population averages toward individualized insight, identifying whether cognitive symptoms relate primarily to sleep fragmentation, stress burden, irregular behavioral rhythms, or symptom-specific factors. Such approaches align with emerging N-of-1 and precision-health paradigms in which individuals serve as their own reference baseline, enabling targeted and adaptive interventions rather than generalized recommendations (Collins and Varmus, 2015; Topol, 2019).

At the same time, potential risks must be acknowledged. Continuous monitoring may inadvertently medicalize normal cognitive variability or promote excessive self-surveillance. Digital biomarkers remain sensitive to device variability, adherence challenges, and contextual noise, and should therefore be framed primarily as hypothesis-generating indicators rather than diagnostic endpoints (Huckvale et al., 2019; Coravos et al., 2019). Economic and sustainability considerations further influence feasibility, as unequal technological access may widen disparities even while scalable monitoring reduces reliance on resource-intensive clinical testing.

Recognizing both opportunities and limitations strengthens a prevention-oriented approach in which technology supports, rather than replaces, clinical judgment and patient-centered care. Positioned in this way, menopause-related brain fog shifts from a source of uncertainty toward an entry point for proactive brain health engagement during midlife.

## 9 Conclusion

Menopause-related brain fog represents a common yet insufficiently operationalized phenomenon situated at the intersection of women's health, cognitive aging, and preventive neuroscience. Rather than viewing these cognitive complaints solely as symptoms requiring confirmation or dismissal, this Perspective proposes that they can be understood as expressions of dynamic brain–body interaction during a time-limited midlife transition. The menopause transition may therefore function as a natural observational window in which variability in cognition, physiology, and daily functioning becomes particularly visible.

Advancing understanding requires a shift from static, deficit-oriented assessment toward ecologically valid measurement capable of capturing within-person change. By integrating patient-centered symptom profiling, targeted cognitive assessment, and longitudinal ecological monitoring through EMA and digital phenotyping, menopause-related brain fog can be reframed as a measurable and scientifically tractable construct (Insel, 2018). Such approaches allow researchers and clinicians to distinguish normative variability from concerning trajectories while identifying modifiable drivers including sleep disruption, stress burden, and behavioral rhythms.

Importantly, this framework extends beyond menopause itself. Transitional life stages characterized by physiological change may provide unique opportunities to study adaptive processes in brain aging more broadly. Emphasizing variability, context, and individualized trajectories aligns with emerging models of precision and preventive neuroscience (Collins and Varmus, 2015; Stern, 2012), shifting attention from late-stage disease detection toward maintenance of cognitive confidence and participation across the lifespan.

At a societal level, reframing menopause-related cognitive complaints may also help reduce stigma and uncertainty surrounding women's midlife brain health. Recognizing brain fog as a meaningful but typically non-neurodegenerative experience supports more constructive clinical dialogue and empowers women to engage proactively with modifiable aspects of health and daily life (Maki and Jaff, 2022).

Ultimately, menopause-related brain fog should not be viewed merely as a transient symptom to be explained away, nor as an early marker of inevitable decline, but as an opportunity to rethink how aging neuroscience measures, interprets, and supports cognitive health in real-world contexts. By embracing ecologically grounded and person-centered approaches, the field may move closer to understanding brain aging as a dynamic process shaped by interaction, adaptation, and prevention rather than loss alone.

## Data Availability

The original contributions presented in the study are included in the article/supplementary material, further inquiries can be directed to the corresponding author.
